# Comment on “Conserved phylogenetic distribution and limited antibiotic resistance of class 1 integrons revealed by assessing the bacterial genome and plasmid collection” by A.N. Zhang et al.

**DOI:** 10.1186/s40168-020-00950-6

**Published:** 2021-01-04

**Authors:** Paul H. Roy, Sally R. Partridge, Ruth M. Hall

**Affiliations:** 1 Centre de Recherche en Infectiologie, CHU de Québec, Québec, QC Canada; 2 Department de Biochimie, de Microbiologie, et de Bio-informatique, Université Laval, Québec, QC Canada; 3 Centre for Infectious Diseases and Microbiology, The Westmead Institute for Medical Research, The University of Sydney and Westmead Hospital, Westmead, New South Wales 2145 Australia; 4 School of Life and Environmental Sciences, The University of Sydney, Camperdown, New South Wales 2006 Australia

**Keywords:** Integron, Integrase, Cassette, XerC, Transposon

## Abstract

An article published in *Microbiome* in July 2018 uses incorrect definitions of integron integrase IntI1 and of class 1 integrons that affect the interpretation of the data.

The article “Conserved phylogenetic distribution and limited antibiotic resistance of class 1 integrons revealed by assessing the bacterial genome and plasmid collection” published in Microbiome [1] describes an “integron integrase database” (File S1) that needs considerable “weeding” and manual curation in order to be useful. The authors use definitions of IntI1 (as opposed to the more general term IntI), and of class 1 integrons, which differ from those used historically and by other researchers in the field and this causes confusion. The criterion “56% to 100%” identity for an IntI to be called IntI1 used in this article is arbitrary and quite simply incorrect. This would include IntI3, which is 61% identical to IntI1. Class 1 integrons are those that encode IntI1 and complete IntI1 proteins are all nearly identical (at least 98% [2]) to that of pDGO100, Tn*21*, and pVS1 [3–5], (WP_000845048). The relevance of the more distantly related IntI to the spread of antibiotic resistance is questionable.

Although it was stated that the entries in the database were curated, this does not appear to be the case. Tyrosine recombinases (integron integrases, phage integrases, XerC, XerD, etc.) must contain the conserved tetrad (Arg…His/Arg-X-X-Arg…Tyr) in order to be functional [6]. Integron integrases possess an additional domain of ca. 30 amino acids relative to other tyrosine recombinases [7]. This domain is involved in single-strand binding in the unique mechanism of recombination used by integron integrases (IntI) [8]. Our database search using a Hidden Markov Model (HMM) of this domain clearly distinguished IntIs from other tyrosine recombinases.

The list of 3383 “integron integrases” in File S1.fasta of Zhang et al. contains fewer than 200 complete IntI1 (> 98% identity to IntI1 of pVS1) and over 700 partial IntI1 sequences and translations of reading frames that extend into adjacent insertion sequences (including 119 that read into IS*26*). The data used for their phylogenetic tree (their Figs. 2 and S1) are flawed and reflect these rearrangements more than it does integrase evolution. One complete integron integrase from *Comamonas thiooxydans*, a beta-proteobacteria [9], is 93% identical to IntI1 of pVS1, and could be called “IntI1-like.”

A Fasta36 (https://github.com/wrpearson/fasta36) comparison of IntI1 with File S1.fasta reveals that, among complete IntIs, the next closest relative of IntI1 (72% identical) is a chromosomally encoded IntI of *Paucibacter* sp. Some chromosomally encoded IntIs of *Rubrivivax* spp. are 68% identical. These chromosomally encoded IntIs are not IntI1, and they are not in class 1 integrons but rather represent novel classes. Another 64 IntIs are > 56% identical to IntI1. Of the 802 entries (including duplicates) with 37-56% identity to IntI1, only 548 are potential IntIs. The 1553 entries with < 37% identity to IntI1 include integrases other than integron integrases (including 78 XerCs with > 95% identity to XerC of *Acinetobacter baumannii*) and even 66 transposases, which are not tyrosine recombinases. The latter may have been included by the use of keywords from annotations that were not identified and corrected. Including entries that are not IntI1, or not even IntI, is not helpful. Use of three filters (length > 280 amino acids, the tetrad of conserved amino acids, and the extra domain of IntIs) would considerably improve the list and discriminate against partial integrases, phage integrases, XerCs, transposases, etc.

Referring to class 1 and 2 integrons found on the chromosome, as opposed to a plasmid, as a “chromosomal integron” also has the potential to cause confusion. Certain bacterial species have integrons on their chromosomes, each with a distinct *intI*, as first described in *Vibrio cholerae* [10]. Different terms have been applied to this type of integron over the years (including the disfavored “superintegron” [11]), but they are currently generally known as “chromosomal integrons” (CI) or “sedentary chromosomal integrons” (SCI) (e.g., Loot et al. [12] ) as opposed to “mobile integrons” (MI) of classes 1, 2, and 3 (and also classes 4 and 5 [13]).

Other questionable definitions used in the article are “In0” that should refer only to the integron in pVS1 that contains the 5′-CS and 3′-CS but no cassettes [5] and “type B” integrons to indicate those having *intI1* but no *sul1* and equating this with being “pre-clinical.” This “type B” would include class 1 integrons with a complete set of transposition genes (*tniABQR*) instead of the 3′-CS, containing *sul1*. Examples include Tn*402* and several integrons encoding carbapenemases such as IMP-9 [14], VIM-2 [15], or IMP-4 [16]. *sul3*-containing class 1 integrons (e.g., [17]) would also be included in “type B.” Although these *tniABQR*-containing integrons and *sul3*-containing integrons appear rarer than *sul1*-containing integrons, they are usually found in clinical isolates.

When discussing resistance genes associated with integrons, Zhang et al. also do not make the important distinction between those carried by gene cassettes and those associated with the integron in other ways. For example, resistance to sulfonamides (Table S8) is conferred by *sul1* in the 3′-CS or *sul3* in the alternative 3′-region of class 1 integrons, while other resistance genes are found with IS*CR1* between partial duplications of the 3′-CS in “complex” class 1 integrons [18–20]. Neither *bla*_TEM_ genes (found in complete or truncated copies of Tn*3* and close relatives [21]), nor tetracycline resistance genes have been found in a gene cassette. Examination of examples of *bla*_TEM_ genes from Table S5 of Zhang et al. suggests that *bla*_TEM_ is part of Tn*1331*, a structure that lacks *intI1* but includes a cassette array inserted within Tn*3* [22], or a derivative (CP018448, CP021778, and FLII01000018), or is located upstream of the 5′-CS (CP006631). From information in Table S10, the *tet* determinant mentioned by Zhang et al. appears to be *tet*(33) found between two copies of IS*6100*, one of which interrupts the 5′-CS (AJ420072) [23].

Zhang et al. also comment on the limited variety of antibiotic resistance genes (ARG) acquired by integrons and possible association of *intI1* with other ARG by co-localization/co-enrichment. It is already well established that each mobile ARG is usually intimately associated with only one particular mobile genetic element (insertion sequence, transposon, gene cassette/integron), responsible for its original capture from a source organism, and that only a subset of ARG are found in gene cassettes [19, 24]. Co-localization of class 1 integrons and other ARG is also already well documented: ARG and associated mobile elements tend to cluster in defined multiresistant regions. Part of this is due to specific associations, such as targeting of the *res* sites of Tn*21*-subfamily by Tn*402*-like transposons/class 1 integrons [20].

Finally, the article’s title and comments on the limited variety of integron-associated ARG imply a diminished role of integrons in antibiotic resistance. The article states that only 60 (page 8) or 69 (page 11) ARG genotypes are associated with class 1 integrons, yet a survey of gene cassettes in 2009 [24] identified 130 different cassettes carrying ARG (< 98% nucleotide identity) at that time, with additional examples being identified in the last 10 years. Genes of the *bla*_VIM_ and *bla*_IMP_ families and some *bla*_GES_ variants, conferring resistance to carbapenemases, are all found in gene cassettes [14–16, 24] clearly indicating that integrons are still of major clinical importance.

If the integron integrase database generated by Zhang et al. were to be filtered, curated, indexed, and made available on a website that also contains the cassette data in Figure 4, it would allow users to clearly see the integrases of dozens of novel classes of chromosomal integrons and provide a useful supplement to existing databases of integrons such as INTEGRALL [25].

## Data Availability

The Fasta36 analysis of IntI1 vs. the proteins in FileS1.fasta of Zhang et al., and the HMM of the additional domain of integron integrases, are available at https://figshare.com/articles/dataset/intI1_fasta36_txt_and_hmmer_intI_rtf/13388069.

## Abbreviations

ARG: Antibiotic resistance gene(s)

## References

[1] Zhang AN, Li LG, Ma L, Gillings MR, Tiedje JM, Zhang T (2018). Conserved phylogenetic distribution and limited antibiotic resistance of class 1 integrons revealed by assessing the bacterial genome and plasmid collection. Microbiome..

[2] Hall RM, Brenner S, Miller JH (2001). Integrons. Encyclopedia of genetics.

[3] Cameron FH, Groot Obbink DJ, Ackerman VP, Hall RM. Nucleotide sequence of the AAD(2”) aminoglycoside adenylyltransferase determinant *aadB*. Evolutionary relationship of this region with those surrounding *aadA* in R538-1 and *dhfrII* in R388. Nucleic Acids Res. 1986;14:8625–35. 10.1093/nar/14.21.8625 PMC311882 3024112

[4] Sundström L, Radström P, Swedberg G, Sköld O (1988). Site-specific recombination promotes linkage between trimethoprim- and sulfonamide resistance genes. Sequence characterization of *dhfrV* and *sulI* and a recombination active locus of Tn*21*. Mol Gen Genet.

[5] Bissonnette L, Roy PH (1992). Characterization of In0 of *Pseudomonas aeruginosa* plasmid pVS1, an ancestor of integrons of multiresistance plasmids and transposons of Gram-negative bacteria. J Bacteriol.

[6] Nunes-Düby SE, Kwon HJ, Tirumalai RS, Ellenberger T, Landy A (1998). Similarities and differences among 105 members of the Int family of site-specific recombinases. Nucleic Acids Res.

[7] Messier N, Roy PH (2001). Integron integrases possess a unique additional domain necessary for activity. J Bacteriol.

[8] MacDonald D, Demarre G, Bouvier M, Mazel D, Gopaul DN (2006). Structural basis for broad DNA-specificity in integron recombination. Nature..

[9] Gillings MR, Boucher Y, Labbate M, Holmes A, Krishnan S, Holley M, Stokes HW (2008). The evolution of class 1 integrons and the rise of antibiotic resistance. J Bacteriol.

[10] Clark CA, Purins L, Kaewrakon P, Manning PA. VCR repetitive sequence elements in the *Vibrio cholerae* chromosome constitute a mega-integron. Mol Microbiol. 1997;26:1137–8. 10.1046/j.1365-2958.1997.d01-5533.x 9426148

[11] Hall RM, Holmes AJ, Roy PH, Stokes HW (2007). What are superintegrons?. Nat Rev Microbiol.

[12] Loot C, Nivina A, Cury J, Escudero JA, Ducos-Galand M, Bikard D, Rocha EP, Mazel D (2017). Differences in integron cassette excision dynamics shape a trade-off between evolvability and genetic capacitance. mBio.

[13] Cambray G, Guerout AM, Mazel D (2010). Integrons. Annu Rev Genet.

[14] Xiong JH, Alexander DC, Ma JH, Déraspe M, Low DE, Jamieson FB, Roy PH (2013). Complete sequence of pOZ176, a 500-kilobase IncP-2 plasmid encoding IMP-9-mediated carbapenem resistance, from outbreak isolate *Pseudomonas aeruginosa* 96. Antimicrob Agents Chemother.

[15] Vilacoba E, Quiroga C, Pistorio M, Famiglietti A, Rodriguez H, Kovensky J, Déraspe M, Raymond F, Roy PH, Centron D (2014). A *bla*_VIM-2_ plasmid disseminating in extensively drug-resistant clinical *Pseudomonas aeruginosa* and *Serratia marcescens* isolates. Antimicrob Agents Chemother.

[16] Xiong JH, Déraspe M, Iqbal N, Jamieson FB, Wasserscheid J, Dewar K, Hawkey PM, Roy PH (2016). Genome and plasmid analysis of *bla*_IMP-4_-carrying *Citrobacter freundii* B38. Antimicrob Agents Chemother.

[17] Moran R, Holt KE, Hall RM (2016). pCERC3 from a commensal ST95 *Escherichia coli*: a ColV virulence-multiresistance plasmid carrying a *sul3*-associated class 1 integron. Plasmid.

[18] Partridge SR, Hall RM (2003). In34, a complex In5 family class 1 integron containing orf513 and *dfrA10*. Antimicrob Agents Chemother.

[19] Arduino SM, Catalano M, Orman BE, Roy PH, Centron D (2003). Molecular epidemiology of orf513-bearing class 1 integrons in multiresistant clinical isolates from Argentinian hospitals. Antimicrob Agents Chemother.

[20] Partridge SR (2011). Analysis of antibiotic resistance regions in Gram-negative bacteria. FEMS Microbiol Rev.

[21] Partridge SR, Hall RM (2005). Evolution of transposons containing *bla*_TEM_ genes. Antimicrob Agents Chemother.

[22] Tolmasky M (2000). Bacterial resistance to aminoglycosides and beta-lactams: the Tn*1331* transposon paradigm. Front Biosci.

[23] Tauch A, Gotker S, Puhler A, Kalinowski J, Thierbach G (2002). The 27.8-kb R-plasmid pTET3 from *Corynebacterium glutamicum* encodes the aminoglycoside adenyltransferase gene cassette *aadA9* and the regulated tetracycline efflux system Tet 33 flanked by active copies of the widespread insertion sequence IS*6100*. Plasmid.

[24] Partridge SR, Tsafnat G, Coiera E, Iredell JR (2009). Gene cassettes and cassette arrays in mobile resistance integrons. FEMS Microbiol Rev.

[25] Moura A, Soares M, Pereira C, Leitão N, Henriques I, Correia A (2009). INTEGRALL: a database and search engine for integrons, integrases and gene cassettes. Bioinformatics..

